# Hydrogenolysis and Activation of Soda Lignin Using [BMIM]Cl as a Catalyst and Solvent

**DOI:** 10.3390/polym9070279

**Published:** 2017-07-12

**Authors:** Shengming Zhang, Liang Liu, Guizhen Fang, Ning Yan, Shixue Ren, Yanli Ma

**Affiliations:** 1 College of Material Science and Engineering, Northeast Forestry University, Harbin 150040, China; zsmdblydx@163.com (S.Z.); liuliang10260211@163.com (L.L.); myl219@126.com (Y.M.); 2 Faculty of Forestry, Department of Chemical Engineering and Applied Chemistry, University of Toronto, Toronto, ON M5S 3B3, Canada; ning.yan@utoronto.ca

**Keywords:** soda lignin, ionic liquid [BMIM]Cl, degradation and activation, reactivity, antioxidant activity

## Abstract

To improve the reactivity of the soda lignin, an acid ionic liquid 1-butyl-3-mthylimidazolium chloride ([BMIM]Cl) was used as the catalyst and solvent to degrade the soda lignin through hydrogenolysis. Structural elucidation of the lignin samples was conducted by using a combination of analytical methods including chemical analysis, ultraviolet spectrophotometry (UV spectrophotometry), Fourier transform infrared spectroscopy (FT-IR spectra), two-dimensional heteronuclear single quantum coherence nuclear magnetic resonance (2D-HSQC NMR) techniques, and gel permeation chromatography (GPC). The antioxidant activities of the lignin samples were evaluated using the diammonium 2,2′-azino-bis(3-ethylbenzothiazoline-6-sulfonate) (ABTS^+^) radical scavenging and 1,1-diphenyl-2-picrylhydrazyl (DPPH) radical scavenging methods. The degradation mechanism was proposed based on the characterization results. The optimal reaction condition was as follows: the concentration of [BMIM]Cl in the solution was 10 wt %, the hydrogen initial pressure was 3 MPa, and the solution was heated for 4 h at 90 °C. After the reaction, the total hydroxyl content of the soda lignin increased by 81.3%, while the phenolic hydroxyl content increased by 23.1%. At the same time, the weight-average molar mass of the soda lignin sample decreased from 8220 to 6450 g/mol with an improved antioxidant activity. In addition, approximately 56.7% of the β-O-4 linkages were degraded in the lreaction. The main effect of the acid ionic liquid [BMIM]C1 was related to the cleavage of β-O-4 linkages. This study has shown the potential of using the catalyzed soda lignin as a natural polymer antioxidant.

## 1. Introduction

As a natural aromatic polymer, lignin has the potential to be used as a raw material for synthesizing functional materials and fine chemicals [1]. For example, it can be used as an additive to improve the performance of adhesives [2,3], a grafting precursor to prepare lignin quaternary ammonium salts [4], and a raw material to produce lignin/polymer (polyvinyl alcohol/polyvinyl chloride) composites [5,6]. Soda lignin is available in large quantities from numerous pulping processes in China. However, a low concentration of active functional groups and a high degree of polymerization are major issues limiting its utilization. Hydrogenolysis is one of the most effective methods to improve lignin’s reactivity [7,8]. In our previous studies, a series of acid and metal catalysts were developed to degrade lignin through hydrogenolysis routes. After the reaction, the reactivity of the soda lignin was greatly improved: A 55.2%–63.0% increase in the total hydroxyl content, a 13.9%–29.4% increase in the content of phenol hydroxyl, and an approximately 10.0% decrease in oxygen content [8,9,10].

Ionic liquid, composed of organic and inorganic cations, is a type of salt that maintains a liquid state at room temperature. Due to their negligible vapor pressure, excellent conductivity, good solubility, non-flammability, and easy recovery, ionic liquids are commonly used as catalysts or solvents for biomass extraction and conversion [11,12,13]. Therefore, some researchers have used ionic liquids for extracting, as well as in the catalytic reactions of lignin and its model compounds. For instance, Xu et al. [14] reported the yield and purity of lignin were improved with pretreatment using the ionic liquid as the solvent. The acid ionic liquids, 1-ethyl-3-methylimidazolium trifluoromethanesulfonate ([EMIM]OTf) and 1-ethyl-3-methylimidazolium chloride ([EMIM]Cl), were both suitable media for Bronsted acid-catalyzed dealkylation of eugenol [15]. Jia et al. [16] found that the β-O-4 bonds in lignin model compounds can react with water in 1-hexyl-3-methylimidazolium chloride ([HMIM]Cl). The molecular weight of oak wood lignin was shown to decrease significantly in the [HMIM]Cl under the mild conditions [17]. Under the acidic conditions, the lignin and its model compound can be acidly degraded, and the stronger acidity theoretically the better for the catalytic reaction.

In our previous work, the self-made catalyst SO_4_^2^^−^/ZrO_2_ solid acid and the metal catalyst CuO/SO_4_^2^^−^/ZrO_2_ were found to degrade and activate soda lignin at 90–120 °C [8,9,10]. Coincidentally, the temperature of ionic liquid [BMIM]Cl used for biomass extraction and conversion was in the range of 75–150 °C [11,18,19]. Therefore, the temperature for [BMIM]Cl-catalysis reaction was similar to what was used in our catalysis reactions. Although [BMIM]Cl is a weak acidic ionic liquid, it is still possible to be used as a catalyst and solvent for activation of lignin. It can also be used together with other metal and acid catalysts. In this study, the soda lignin, extracted from the wheat straw black liquor, was catalytically degraded by using an acidic ionic liquid [BMIM]Cl. The optimum reaction conditions were determined by a single factor experiment using the total and phenolic hydroxyl contents as the index. Chemical analysis, UV spectrophotometry, FT-IR spectra, 2D-HSQC NMR techniques, and GPC were utilized to identify the structural features of lignin before and after reactions. Additionally, DPPH radical scavenging and ABTS^+^ radical scavenging measures were used to evaluate lignin’s antioxidant activity. Based on the analysis results, the reaction products from the lignin degradation processes were discussed.

## 2. Materials and Methods

### 2.1. Materials

The black liquor dry matter, which was separated from the wheat straw pulp, was used to produce the soda lignin (SL) following the acid precipitation method [8]. The yield of soda lignin was approximately 15% by weight. The weight content of acid-soluble lignin, acid-insoluble lignin, total sugars, and ash in soda lignin were 2.9%, 89.3%, 3.2% and 4.6%, respectively [8]. The weight content of carbon, oxygen, nitrogen, hydrogen, and sulfur were 56.7%, 26.9%, 3.5%, 5.8% and 2.5%, respectively [8]. The soda lignin had a purity of 92.2%. [BMIM]Cl (1-butyl-3-methylimidazolium chloride, 99%) was provided by Shanghai Chengjie Chemical Corporation (Shanghai, China).

### 2.2. Degradation of Soda Lignin

Different weighted amounts of [BMIM]Cl were added into a dioxane–water (9:1, *v*/*v*) solution to prepare mixed solutions with the ionic liquid weight concentration of 4%, 6%, 8%, 10% and 12%, respectively. Three grams of soda lignin were dissolved in a mixed solution of totaling 200 mL in volume. Then, the mixture was transferred to a reaction kettle (1-L, Dalian-Controlled Plant Company, Dalian, China). The reactor was filled with 3 MPa hydrogen and the reaction was then started. Under the reaction condition of [BMIM]Cl concentration of 4 wt % at 100 °C, the effect of time was investigated in 2, 4 and 6 h. After that, the reaction was carried out at 70, 80, 90, 100 and 110 °C, respectively. Finally, the effect of the concentration of the ionic liquid was investigated. After the reaction was completed, the dioxane solution was removed using a rotary evaporator. One liter of 50 °C of distilled water was added to the remaining mixture. Then, the mixture was decompression filtered. The filter cake was dried at 45 °C for 24 h with a vacuum of 0.08 MPa to obtain the catalyzed soda lignin; furthermore, the distilled water in the filtrate can be removed by the rotary evaporator to obtain the recovered ionic liquid, but about 3.0% by weight of the soda lignin remained in the recovered ionic liquid.

### 2.3. Characterization of Products

Chemical titration was used to measure the total hydroxyl content of the lignin samples, as described in [8]. The acetylated lignin sample was titrated using an NaOH solution and the total hydroxyl value was then calculated. The phenolic hydroxyl content of the lignin samples was measured through the ultraviolet-spectroscopy method [8,20]. The FT-IR spectra of the lignin samples were captured by a Nicolet Nexus 670 FT-IR spectrometer (Nicolet Instrument Co., Madison, WI, USA). The scanning range was from 4000 to 500 cm^−1^ at a resolution of 4 cm^−1^ with 16 scans. A Bruker-AVIII-400 MHz spectrometer (Bruker Co., Fällanden, Switzerland) was used to record the nuclear magnetic resonance (NMR spectroscopy) images at 25 °C in DMSO-d_6_. A portion of 0.5 mL of DMSO-d_6_ was used to dissolve 25 mg of lignin for quantitative ^1^H–NMR experiments. For the quantitative ^13^C NMR experiments, 0.5 mL of DMSO-d_6_ was used to dissolve 140 mg of lignin. The FT mode was used to record the quantitative spectra at 100.6 MHz. For the 2D-HSQC experiments, 0.5 mL was DMSO-d_6_ was used to dissolve 60 mg of lignin. HSQC experiments were performed with the Bruker standard pulse program hsqcetgpsi2, with some parametric modification. Commonly used parameters were chosen, which can be found elsewhere [21]. The Topspin NMR software was used for data processing. An Agilent 1100 high-performance liquid chromatography apparatus was used to measure the molecular weight of the acetylated lignins, as described in [8].

### 2.4. Antioxidant Activity

ABTS^+^ radical (ABTS^+^•) and DPPH radical (DPPH•) scavenging assays were performed using the method described in [10]. For the DPPH• assay, different concentrations of lignin samples were prepared by using the dioxane–water as a solvent. The lignin solution of 1 mL was then added to the DPPH ethanol solution of 4 mL. The mixture was shaken and allowed to stand for 30 min. In addition, a mixture of dioxane–water (1 mL) and DPPH ethanol solution (4 mL) was used as the contrast. Based on the absorbance at 517 nm, the DPPH• scavenging rate of the lignin samples was calculated. The formula is given as follows:

(1) $$\mathrm{Scavenging}\;\mathrm{rate}\;\left(\%\right)=\left(A_{contrast}-\;A_{sample}\right)÷A_{contrast}\times 100$$

*A_contrast_* is the absorbance value of the contrast; *A_sample_* is the absorbance value of the lignin sample.

For the ABTS^+^• assay, the ABTS^+^• stock solution of 7.4 mM was prepared using phosphate buffer of 2.6 mM and allowed to stand for 12–16 h. The ABTS^+^• stock solution was added to the phosphate buffer, while the absorbance value set at 0.700 (±0.050) at 25 °C through adding the phosphate buffer. The mixture was recorded as the ABTS^+^• solution. Then, the lignin solution of 0.1 mL was added to the ABTS^+^• solution of 4.9 mL in volume. After 10 min, the ABTS^+^• scavenging rate of lignin was calculated based on the absorbance at 734 nm. A mixture of phosphate buffer of 0.1 mL and ABTS^+^• solution of 4.9 mL was chosen as the contrast. The calculation for the scavenging rate is the same as what was used for DPPH• scavenging rate given in Equation (1).

## 3. Results

### 3.1. Degradation and Activation of Soda Lignin

**Figure 1:**
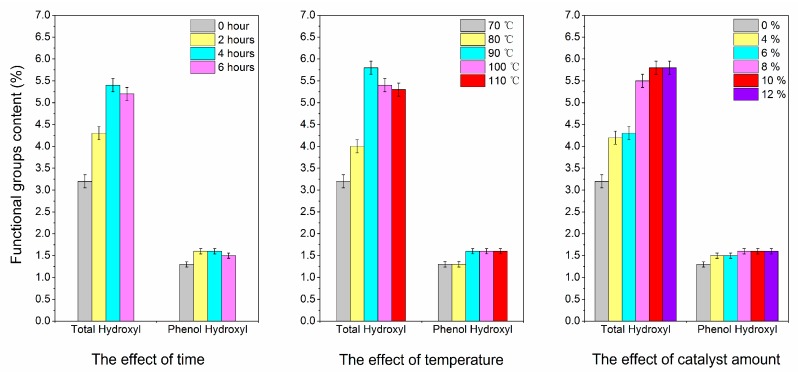
The effects of reaction factors on degradation reaction.

The effects of reaction conditions on lignin degradation are shown in Figure 1. All reactions were carried out under the 3 MPa of hydrogen pressure. The effect of reaction time was observed as follows: the concentration of [BMIM]Cl in the solution was set to be 10 wt %, and the system was then heated at 100 °C. From 0 to 4 h, the total hydroxyl content increased gradually. When the reaction time reached 4 h, the total hydroxyl content attained the maximum, which was 5.4 mmol/g. The total hydroxyl content showed almost no increase for the next 2 h. Furthermore, when the reaction time reached 2 h, the content of phenolic hydroxyl attained the maximum value of 1.6 mmol/g. When the reaction time was over 4 h, the phenolic hydroxyl content showed no increased. Therefore, the optimal reaction time was found to be 4 h.

The effect of temperature was shown as follows: the concentration of [BMIM]Cl in the solution was fixed at 10 wt %, and the system was heated for 4 h starting from the room temperature. When the temperature was below 70 °C, the total hydroxyl content showed no increase. As the reaction temperature raised to 90 °C, the total hydroxyl content reached the maximum value of 5.8 mmol/g. When the reaction temperature was between 100 and 110 °C, the increase in the total hydroxyl was less than what was at 90 °C. Furthermore, when the temperature reached 90 °C, the content of phenolic hydroxyl attained the maximum value of 1.6 mmol/g. Thus, the optimal reaction temperature was controlled at 90 °C.

To investigate the effect of [BMIM]Cl concentration, we performed reaction at 90 °C for 4 h. The content of total hydroxyl groups increased gradually with the increasing concentrations of the [BMIM]Cl solution. When the [BMIM]Cl concentration attained 10 wt % in the solution, the content of total hydroxyl reached the maximum value of 5.8 mmol/g. Meanwhile, the content of phenolic hydroxyl attained the maximum value of 1.6 mmol/g when the concentration of [BMIM]Cl reached 8 wt % in the solution. With the ionic liquid’s concentration was increased, the phenolic hydroxyl content remained unchanged. Therefore, the optimum ionic liquid concentration was selected to be at 10 wt %.

Therefore, the optimal reaction condition was found to be as follows: the system was filled with hydrogen with an initial pressure of 3.0 MPa, the concentration of [BMIM]Cl in the solution was 10 wt %, and the system was allowed to react at 90 °C for 4 h. As a result, the total hydroxyl content of lignin sample after the reaction increased by 81.3%, and the phenolic hydroxyl content of that was increased by 23.1%. The yield of Catalyzed-SL was approximately 81.2% by weight, except about 3.0% by weight of the soda lignin remaining in the recovered ionic liquid.

### 3.2. Characterization of Products

**Figure 2:**
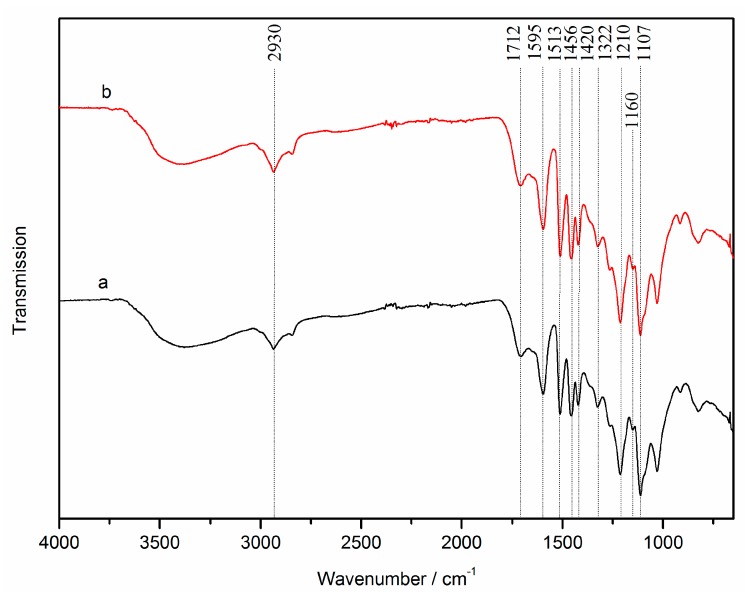
FT-IR spectra of SL (**a**) and Catalyzed-SL (**b**).

The FT-IR spectra of SL and Catalyzed-SL are shown in Figure 2. The conjugated C=O in ester groups of G-S-H lignin was observed at the peak of 1160 cm^−1^. The type of SL and Catalyzed-SL were G-S-H typical. The broad peaks from 3200 to 3600 cm^−1^ were assigned to the stretch in OH groups. The absorption at 1595 and 1513 cm^−1^, which obtained in both spectra, were attributed to the benzene ring skeleton vibration. The absorption at 1420 and 1322 cm^−1^ were the lignin’s characteristic absorption peak. The peak at 2930 cm^−1^ in the spectra was attributed to the CH stretch in the CH_2_ and CH_3_ groups. The peak at 1712 cm^−1^ was observed due to the carbonyl stretching vibration. The bending vibration absorption of methyl was observed at 1456 cm^−1^. Due to the C–H bending vibration, the peaks at 1210 and 1107 cm^−1^ were observed. The infrared spectrum was consistent with what was reported by Faxi [22] and Baddi et al. [23]. Compared with SL, the structure of phenyl–propane skeleton of Catalyzed-SL was stable, and the vibration intensity of some other peaks in the lignin was unchanged. Therefore, the reactions did not degrade the C_9_ basic unit of lignin.

**Figure 3:**
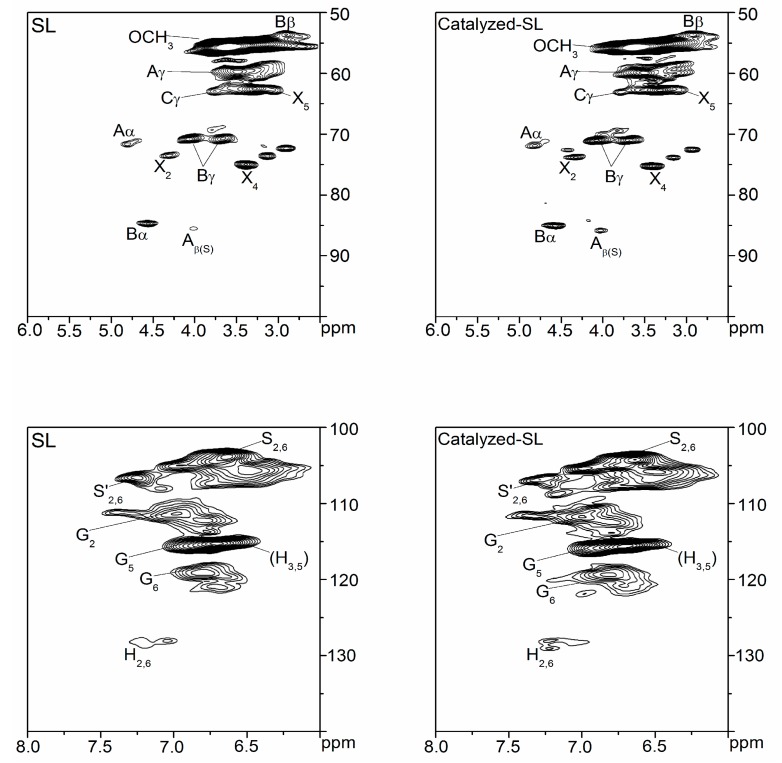
2D-HSQC NMR spectra of lignin samples.

The 2D-HSQC spectra of SL and Catalyzed-SL are depicted in Figure 3. Based on recent studies [8,24,25], the detailed assignments of ^13^C–^1^H cross signals are determined. The signals of lignin side chain region appear in the range of δ_C_/δ_H_ 50.0/6.00 to δ_C_/δ_H_ 100.0/2.50 ppm. In this region, the signals of A_α-G_ at δ_C_/δ_H_ 71.5/4.77 ppm, A_α-S_ at 72.3/4.90 ppm, A_β-G_ at 84.0/4.32 ppm, A_β-S_ at 86.4/4.15 ppm, and A_γ_ at 59.8/3.49 ppm can be explained by the β-O-4 linkage (A). Meanwhile, the signals for β-β linkage (B) shown at δ_C_/δ_H_ 54.0/3.10 ppm (C_β_–H_β_), δ_C_/δ_H_ 85.3/4.70 ppm (C_α_–H_α_), δ_C_/δ_H_ 71.4/4.22 ppm(C_γ_–H_γ_), and δ_C_/δ_H_ 71.4/3.85 ppm (C_γ_–H_γ_). The signals in the range of δ_C_/δ_H_ 100.0/8.00 to δ_C_/δ_H_ 140.0/6.00 ppm can be used to determine the aromatic rings of lignin units. The signal of C_2_–H_2_ at δ_C_/δ_H_ 104.3/6.73 ppm was assigned to the S unit. The signals of C_2,6_ and C=O at δ_C_/δ_H_ 106.6/7.30 ppm can be attributed to the S units (S_2,6_’). G units were found with signals at δ_C_/δ_H_ 111.3/7.03 ppm (C_2_–H_2_), 119.3/6.80 ppm (C_6_–H_6_), 115.2/6.74 ppm (C_5_–H_5_), and 115.2/6.98 ppm (C_5_–H_5_). The signal for H units (C_2,6_–H_2,6_) was observed at δ_C_/δ_H_ 127.7/7.17 ppm.

After the main signal attribution was determined, we used the ^13^C–NMR and 2D-HSQC spectra combination method to quantify the major inter-unit linkages and structural units. The method can be found elsewhere [24,26]. The amounts of β-O-4 linkages in RL and Catalyzed-SL were 4.16/100 Ar and 1.80/100 Ar, respectively. The contents of β-β linkages in RL and Catalyzed-SL were 4.20/100 Ar and 4.95/100 Ar, respectively. The results suggested that approximately 56.7% of the β-O-4 linkages were degraded in the reaction. Meanwhile, the S/G ratios of SL and Catalyzed-SL were determined to be 1.47 and 1.47. Thus, the reduction reaction did not change the lignin’s S/G ratio.

**Figure 4:**
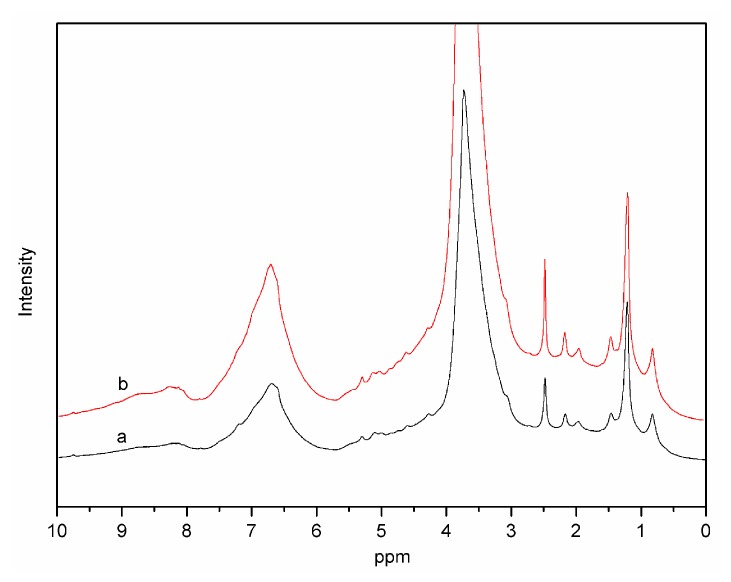
The ^1^H–NMR spectra of SL (**a**) and Catalyzed-SL (**b**).

In addition, the ^1^H–NMR spectra of SL and Catalyzed-SL were depicted in Figure 4. The content of OCH_3_ group of lignin samples was determined based on ^1^H–NMR spectra [27]. The methoxyl contents of SL and Catalyzed-SL were 6.9 and 6.8 mmol/g, respectively. There was no obvious change between SL and Catalyzed-SL. We have already shown that the S/G ratio of lignin had almost no change during the reaction. Therefore, we believe that the methoxy group on the lignin’s aromatic ring did not degrade.

Table 1 shows the molecular weight of SL and Catalyzed-SL. A decrease from 8220 to 6450 g/mol was shown for *M*_W_ and a decrease from 3120 to 2150 g/mol was shown for *M*_n_. The soda lignin was shown to be degraded after the catalyzed reaction.

### 3.3. Antioxidant Activities

**Figure 5:**
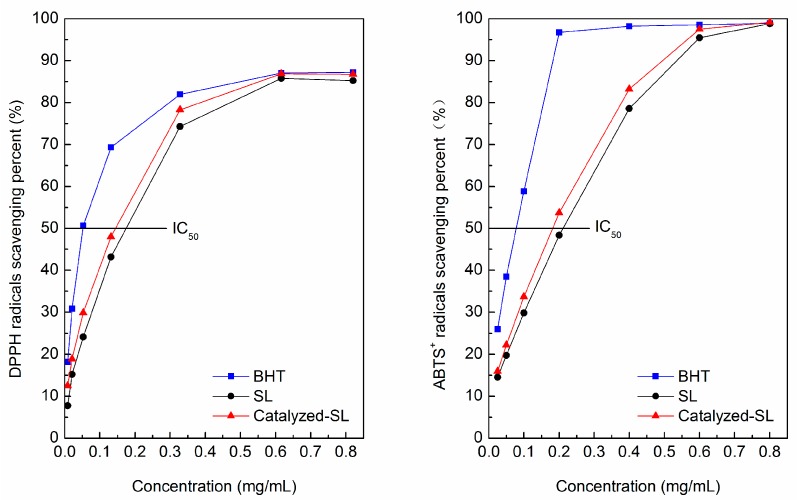
DPPH radicals and ABTS^+^ radicals scavenging percent of lignin samples.

Figure 5 shows the DPPH• and ABTS^+^• scavenging activity of the lignin samples. A common antioxidant BHT (butylated hydroxytoluene) was used as a reference. IC_50_ indicates the half maximal inhibitory concentration of the free radicals. Lower IC_50_ values result from the higher antioxidant activities [28]. For the DPPH• scavenging method, lignin samples had a lower IC_50_ value in the Catalyzed-SL (143.9 mg/mL), which was decreased by 17.2% compared with SL (173.7 mg/mL). Aadil et al. [29] reported the higher phenolic hydroxyl content led to a stronger antioxidant capacity of lignin. Ugartondo et al. [30] and Dizhbite et al. [31] found that the lignin with a lower molecular weight usually had a better antioxidant capacity. In comparison with SL, the Catalyzed-SL had a higher content of phenolic hydroxyl and lower molecular weight, which led to its stronger antioxidant activity. For the ABTS^+^• scavenging method, lower IC_50_ values were observed in the Catalyzed-SL (180.1 μg/mL), which was less than SL (210.0 μg/mL) by 14.2%. Arshanitsa et al. [32] and Thana et al. [33] found that the ABTS^+^• scavenging mechanism can be dependent on the electron or proton transfer. The Catalyzed-SL had higher total hydroxyl content and better reactivity than SL, which means it has a better ABTS^+^• scavenging ability. In summary, the soda lignin’s antioxidant ability was improved after the reaction. Although the lignin samples had a low antioxidant capacity as that of BHT, as natural polymer compounds, they showed improvement after the catalyzed reactions.

## 4. Discussion

The Quantitative 2D–NMR analysis showed that the β-O-4 linkages content of the catalyzed soda lignin was decreased from 4.16/100 to 1.80/100 Ar. In natural lignin, the β-O-4 bond is about 50% of the total number of all chemical bonds [1]. Compared with the value of 50%, the measured content of β-O-4 linkages of the soda lignin was significantly lower. This phenomenon is because most of the β-O-4 linkage has been already cleaved through the treatment of strong base in the soda pulping process of papermaking. Even so, there is still about 56.7% of the β-O-4 linkages degraded in reaction. In GPC measurements, the *M*_w_ of soda lignin after the catalyzed reaction was reduced from 8220 to 6450 g/mol and the *M*_n_ was reduced from 3120 to 2150 g/mol. The FT-IR analysis showed that the aromatic structure of the catalyzed-soda lignin was stable. Moreover, we obtained a lignin yield of up to 81.2%, which means that the phenomenon of benzene ring cracking most likely did not occur. The degradation is mainly due to β-O-4 linkage breakage. In the Quantitative 2D–NMR, the lignin’s S/G ratio did not change during the reaction. The ^1^H–NMR quantification further showed that the value of OCH_3_ on lignin’s aromatic ring did not change, which means that the OCH_3_ on the lignin’s aromatic ring did not degrade, and the S units did not convert to G units in the reaction. In the determination of the antioxidant activity, the catalyzed soda lignin’s antioxidant activity was significantly improved. It has been shown that lignin with a higher antioxidant activity usually contains more phenolic hydroxyl groups than lignin with a lower antioxidant activity [10]. Since the increase in the hydroxyl value was confirmed by the chemical analysis, we believe that new hydroxyl groups had most likely occurred at the C_4_ position on the aromatic ring after the β-O-4 linkage cleavage. Future work could consider further analysis of different types of –OH groups using ^31^P NMR technique.

Under the acidic conditions, the lignin can be hydrolyzed to form the Hibbert ketones [34]. Mitchell et al. [35] analyzed the hydrolysate produced by dilute acid pretreatment of the biomass feedstock and found that the phenolic compounds were dominated by the Hibbert ketones. Deuss et al. [36] found that the products of the lignin model compound after acid hydrolysis by the triflic acid was essentially the Hibbert ketones (C_3_-monomers). Jia et al. studied the degradation of lignin’s model compound by [BMIM]Cl and they found that the β-O-4 linkage was degraded [16]. In this study, the soda lignin may be acid hydrolyzed and the remaining β-O-4 linkages in the soda lignin were cleaved, resulting in the formation of lignin-bound Hibbert ketones structure. This phenomenon leads to a strong increase in total hydroxyl groups of the catalyzed soda lignin.

**Figure 6:**
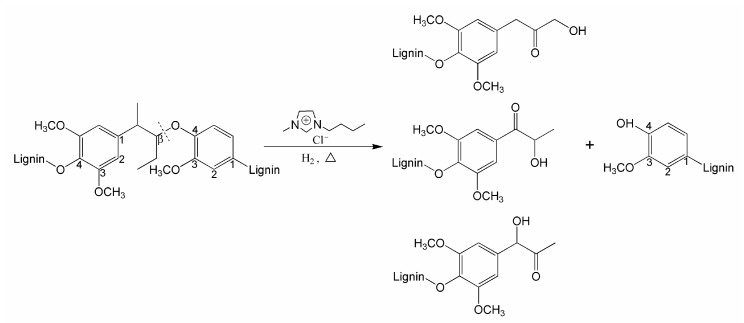
Possible reaction products from the β-O-4 linkages cleavage.

In our previous studies, a solid acid catalyst SO_4_^2−^/ZrO_2_ was proven to be useful for degradation of the soda lignin through the hydrogenolysis route. [8]. In the activation reaction, the Bronsted acid sites provided the active center [9]. Chiappe et al. found that the [Hmim]^+^ cation had a similar effect as Bronsted acids [37]. Therefore, we suggest that the effect of the [BMIM]^+^ cation is likely to be similar to Bronsted acid. The [BMIM]^+^ cation could provide catalytic activity in lignin activation reactions. In summary, the main effect of the acid ionic liquid [BMIM]C1 is to break down the β-O-4 linkages in the soda lignin, and the newly formed lignin-bound Hibbert ketones structure results in a significant increase in total hydroxyl groups. The possible reaction products are depicted in Figure 6.

## 5. Conclusions

Soda lignin degradation and activation were conducted through hydrogenolysis with the usage of an acid ionic liquid [BMIM]Cl. The optimal reaction condition was found to be as follows: the concentration of [BMIM]Cl in the solution was 10 wt %, an initial system pressure of 3 MPa with hydrogen, and a reaction temperature of 90 °C and a reaction time of 4 h. Compared with the original soda lignin, the amount of total hydroxyl content in the catalyzed lignin was increased by 81.3%, while the phenolic hydroxyl content of the catalyzed lignin was increased by 23.1%. Meanwhile, the weight-average molecular weight of the soda lignin reduced from 8220 to 6450 g/mol. After catalytic reaction, the soda lignin’s antioxidant activity was improved. Approximately 56.7% of the β-O-4 linkages were degraded in the reaction, whereas lignin’s S/G ratio and the OCH_3_ content remained almost unchanged. Lignin degradation can be explained by the cleavage of β-O-4 linkages. The newly formed lignin-bound Hibbert ketones structure results in a significant increase in total hydroxyl groups. The main effect of the acid ionic liquid [BMIM]C1 was to cleave the β-O-4 linkages in the soda lignin samples.

## Figures and Tables

**Table 1:** Molecular weight distribution of lignin samples.

Sample	*M*_w_	*M*_n_	*M*_w_/*M*_n_
SL	8220	3120	2.63
Catalyzed-SL	6450	2150	3.00
